# Baseline Characteristics Associated with Improved Outcomes in Patients Undergoing Primary Decompressive Craniectomy for Acute Subdural Hematoma Evacuation—A Retrospective Observational Study

**DOI:** 10.3390/medicina61020288

**Published:** 2025-02-07

**Authors:** Rimantas Vilcinis, Raimondas Juskys, Lukas Piliponis, Arimantas Tamasauskas

**Affiliations:** 1 Department of Neurosurgery, Faculty of Medicine, Medical Academy, Lithuanian University of Health Sciences, 44307 Kaunas, Lithuaniaarimantas.tamasauskas@lsmu.lt (A.T.); 2 Institute of Anatomy, Faculty of Medicine, Medical Academy, Lithuanian University of Health Sciences, 44307 Kaunas, Lithuania; 3 Neuroscience Institute, Medical Academy, Lithuanian University of Health Sciences, 44307 Kaunas, Lithuania

**Keywords:** acute subdural hematoma, primary decompression, decompressive craniectomy, outcomes, cisternal effacement score

## Abstract

*Background and Objective*: The study’s aim is to identify a subgroup of patients who would benefit from primary decompressive craniectomy (pDC) after acute subdural hematoma (aSDH) evacuation. *Materials and Methods*: A retrospective analysis of 290 patients undergoing aSDH evacuation between 2016 and 2021 was conducted. Osteoplastic craniotomy (OC) was performed in 213 cases (73.4%), whereas 77 individuals underwent pDC. Preoperative characteristics, such as age, initial GCS score, hematoma thickness, midline shift, and cisternal effacement score (CES), were used to predict outcome at discharge by the Glasgow Outcome Scale (GOS). *Results*: Older age, lower initial GCS, and higher CES preoperatively were independently associated with lower GOS scores at discharge. Age and degree of cisternal compression remained significant predictors of GOS score in the pDC subgroup. Survivors who underwent pDC were younger in comparison to deceased individuals receiving OC (mean age 55.43 ± 14.58 vs. 72.28 ± 14.63, *p* < 0.001). Patients who achieved favorable outcomes after pDC were significantly younger compared to those who had poor outcomes after OC (mean age 49.20 ± 12.05 vs. 72.28 ± 14.32, *p* < 0.001). *Conclusions*: Younger patients (<55 years old) with initial GCS scores of 4–6, midline shifts of 1 to 2 cm, subdural hematoma thickness of 1 to 2.5 cm, and CES in a range of 7–12 may benefit from pDC as it could potentially improve survival and functional outcomes after aSDH evacuation.

## 1. Introduction

Traumatic brain injury (TBI) is a significant global public health issue, and one of its critical components is intracranial hypertension (IHT), especially when it persists and is not alleviated in a timely manner. Prompt evacuation of intracranial hematoma and the relief of brain compression are crucial to prevent extensive cerebral damage. Removal of the hematoma volume can often obviate the need for further surgical treatment. However, if the dura mater cannot be closed without tension after hematoma evacuation, primary decompressive craniectomy (pDC) becomes necessary [1].

Secondary DC, although effective in reducing intracranial pressure (ICP) and improving survival rates, is associated with a higher rate of disability and various complications [2]. These include cerebrospinal fluid circulation disorders such as subdural effusions and hydrocephalus, wound healing complications, purulent infections, and, in rare cases, syndrome of the trephined [2,3,4]. Additionally, there are also risks associated with cranioplasty surgery, which follows decompression several months after cranial bone flap removal [5]. Secondary DC performed in response to post-operative edema as a last-resort therapy to control medically refractory IHT represents a clinically distinct situation in comparison to pre-emptive pDC, where decompression is performed in anticipation of post-operative IHT.

Currently, the decision on whether to replace the craniotomy bone flap following hematoma evacuation is primarily based on the neurosurgeon’s judgment. A retrospective cohort study showed that craniotomy for acute subdural hematoma (aSDH) is performed ten times as often as primary DC in the United States [6]. In contrast, a survey involving European, British, and Irish neurosurgeons has shown that 44% of European practitioners opt for primary DC in over half of all aSDH cases, as compared to only 21% of British and Irish counterparts. Furthermore, there was limited agreement within departments, as only 26% of neurosurgical units with at least two respondents demonstrated intra-departmental agreement regarding when to use primary DC for evacuation of aSDH [7,8]. These findings highlight a substantial variation in practice both between different developed countries and even within the individual departments, emphasizing the lack of clear scientific guidance pertaining to the topic.

This study examines how pre-existing baseline characteristics affect the outcomes after osteoplastic craniotomy (OC) and pDC. The goal of the study is to assess when pDC may be more beneficial than OC for patients undergoing the removal of aSDH. The study emphasizes the significance of considering pre-operative variables in selecting a surgical approach and provides insights into the factors that could influence the decision between OC and pDC for aSDH evacuation.

## 2. Materials and Methods

A retrospective analysis was conducted on 290 adult patients after TBI who underwent acute subdural hematoma (aSDH) evacuation at the Department of Neurosurgery of the Lithuanian University of Health Sciences Hospital in Kaunas between 2016 and 2021. Lithuanian University of Health Sciences Hospital Kaunas Clinics is a tertiary university hospital that provides emergency and elective neurosurgery services. The hospital serves the central and partly western regions of Lithuania containing ~1.5 million population. The neurosurgical department performs ~75–100 emergency cases for subdural hematomas and other intracranial bleeds every year. The patients in the sample were selected from the prospectively maintained database of brain-injured individuals who were treated surgically with craniotomy for aSDH and had pre- and post-operative CT images available. The surgical procedures involved the early evacuation of subdural hematoma followed by either OC (*n* = 213) or pDC (*n* = 77). Notably, children and pregnant women were not included in the study cohort. The choice of surgical technique was based on the operating neurosurgeon’s evaluation of the patient’s clinical and intra-operative data, as well as their personal experience.

The collected data include gender, age, the initial GCS score, maximal hematoma thickness, pre- and post-operative midline shift, the degree of cisternal compression before and after surgery, and outcomes. Radiological measurements were assessed based on the pre-operative and 6 h post-operative CT scans. The displacement of the septum pellucidum from the midline on axial images was used to calculate the midline shift. Outcomes were assessed using the Glasgow Outcome Scale (GOS) at the time of discharge from the hospital. Results were dichotomized according to mortality (GOS 1 vs. GOS 2–5) and an unfavorable neurological outcome (GOS 1–2 vs. GOS 3–5).

**Figure 1:**
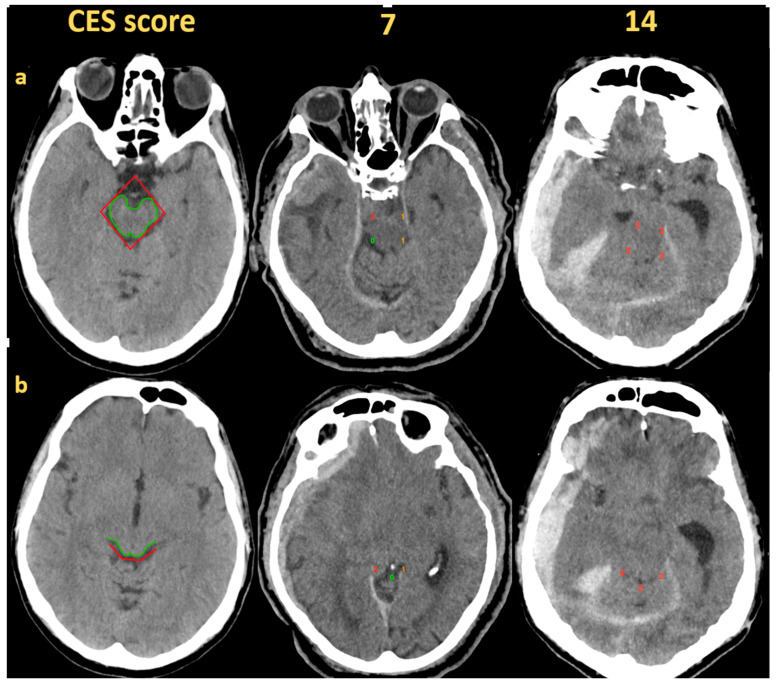
Cisternal effacement score evaluation is based on CT axial cuts at the level of the perimesencephalic (upper row, **a**) and quadrigeminal cisterns (lower row, **b**). The areas marked with the red color represent cisternal components used to calculate the cisternal effacement score. Two cases with CES of 7 and 14 are presented as an example.

The extent of cisternal compression was analyzed by utilizing the cisternal effacement score (CES), which consists of two cisternal components: the perimesencephalic segment located at the level of dorsum sellae and the quadrigeminal segment positioned at the level of tectum [9]. The perimesencephalic cistern was divided into four sections resembling a rhomboid shape, while the quadrigeminal cistern had three parts that were shaped like a semilunar form. The CES calculation methodology and illustrative cases are described in Figure 1. Each of the seven segments was assigned a score ranging from 0 to 2. A score of 0 indicated no compression, a score of 1 indicated partial effacement and a score of 2 indicated complete obliteration of the respective cistern. Therefore, the total CES ranged from 0 to 14 [9].

Ethical approval for this study was obtained from the Kaunas Regional Biomedical Research Ethics Committee (No. BE-2-105, renewed in 2024) and is in accordance with the ethical research principals of the Declaration of Helsinki.

### Statistical Data Analysis

The statistical analysis was performed using R Project for Statistical Computing (Version 4.3.1). Descriptive statistics, hypothesis testing, and regression methods were employed to analyze the data. Summary measures of central tendency were reported as mean ± standard deviation (SD) for parametric and median with interquartile range (IQR) for non-parametric variables. The normality of the data was assessed using the Kolmogorov–Smirnov test, and the variance was evaluated using Bartlett’s test. A significance level of 0.05 was used for hypothesis testing, and *p*-values below this threshold were deemed statistically significant in two-sided analyses. All reported results were rounded to two decimal places.

For normally distributed variables with equal variances, the means between two groups were compared using Student’s *t*-test, whereas Welch’s one-way ANOVA was used for comparing means among several groups. Similarly, skewed data were analyzed using the Mann–Whitney U test for comparisons between two groups and the Kruskal–Wallis ANOVA for comparisons among several groups. The Holm–Bonferroni correction was applied for respective comparison tests to determine specific pairwise differences between GOS groups.

The relationship between baseline characteristics and ordinal GOS scores was examined using both univariate and multivariate proportional odds logistic regression methods. All significant predictors from the univariate analysis were included in the multivariate model, and the goodness-of-fit was assessed using the Lipsitz test.

## 3. Results

### 3.1. Sample Characteristics

The study enrolled 290 patients with a mean age of 63.5 ± 16.5 years who underwent aSDH evacuation. Most of the samples were male (69%, *n* = 200), and they were significantly younger than females (mean age = 60.1 ± 15.8 vs. 71.1 ± 15.6, *p* <0.001). OC was performed for 213 patients (73.4%), while 77 individuals (26.6%) underwent pDC. The pDC group had a lower mean age (59.6 ± 17.6 vs. 64.9 ± 15.9, *p* = 0.02), lower pre-operative Glasgow Coma Scale (GCS) score (median of 4 [3–5] vs. 10 [5–14], *p* < 0.001), and a higher degree of pre-operative midline shift (mean 15.4 ± 6.8 mm vs. 10.2 ± 6.9 mm, *p* < 0.001) compared to the OC group. Furthermore, the pDC group had worse status of basal cisterns both pre- and post-operatively (pre-operative cisternal effacement score median = 11 [8–13] vs. 4 [2–9], *p* < 0.001; post-operative cisternal effacement score median = 4 [2–11] vs. 2 [0–4], *p* < 0.003). However, there was no statistically significant difference in hematoma thickness between the two groups (18.2 ± 6.3 vs. 20.1 ± 7.3, *p* = 0.052). The main sample characteristics are summarized in Table 1.

### 3.2. Analysis of Outcomes

**Figure 2:**
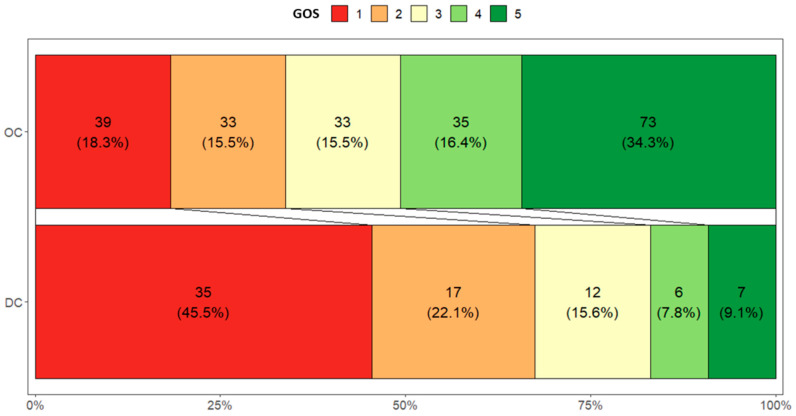
Distribution of GOS scores at hospital discharge among patients undergoing OC and primary DC for aSDH evacuation.

The overall intrahospital mortality rate was observed to be 25.5% (*n* = 74). Similarly, 42.8% of cases (*n* = 124) resulted in an unfavorable outcome. Upon subgroup analysis, the mortality rate was found to be 18.3% (*n* = 39) for the osteoplastic craniotomy (OC) group and 45.5% (*n* = 35) for the primary decompressive craniectomy (pDC) group. Furthermore, an unfavorable neurological outcome at discharge was observed in 33.8% (*n* = 72) of the OC group and 67.5% (*n* = 52) of the pDC group. The summary of the frequency of outcomes is presented in Figure 2.

### 3.3. Baseline Characteristics Associated with the GOS Outcome Groups

The relationship between pre-operatively available variables (age, initial GCS, hematoma thickness, midline shift, and CES) and GOS outcome categories was investigated by employing the ordinal logistic regression method. In both the overall sample and OC group alone, older age, lower initial GCS score, larger hematoma thickness accompanied by greater midline shift, and worse pre-operative state of CES were all found to be associated with lower GOS scores in the univariate analysis. However, within the pDC group alone, only age, hematoma thickness, and degree of cisternal compression were significant predictors of GOS scores. Results of the univariate analysis are detailed in Table 2.

A multivariate proportional odds logistic regression analysis was performed to identify the independent predictive factors of the pre-operatively collected variables on the GOS outcome categories. After adjusting for baseline characteristics, it was found that older age (OR [95% CI] = 0.96 [0.93–0.96]), lower initial GCS (OR [95% CI] = 1.29 [1.20–1.39]), and worse state of basal cisterns preoperatively (OR [95% CI] = 0.85 ([0.79–0.92]) were independently associated with lower GOS scores at discharge for both the entire sample and OC subgroup. Notably, GCS on admission did not reach statistical significance (OR [95% CI] = 1.08 [0.90–1.30]) in the analysis for the pDC group alone. A summary of the multivariate assessment results is provided in Table 3.

**Figure 3:**
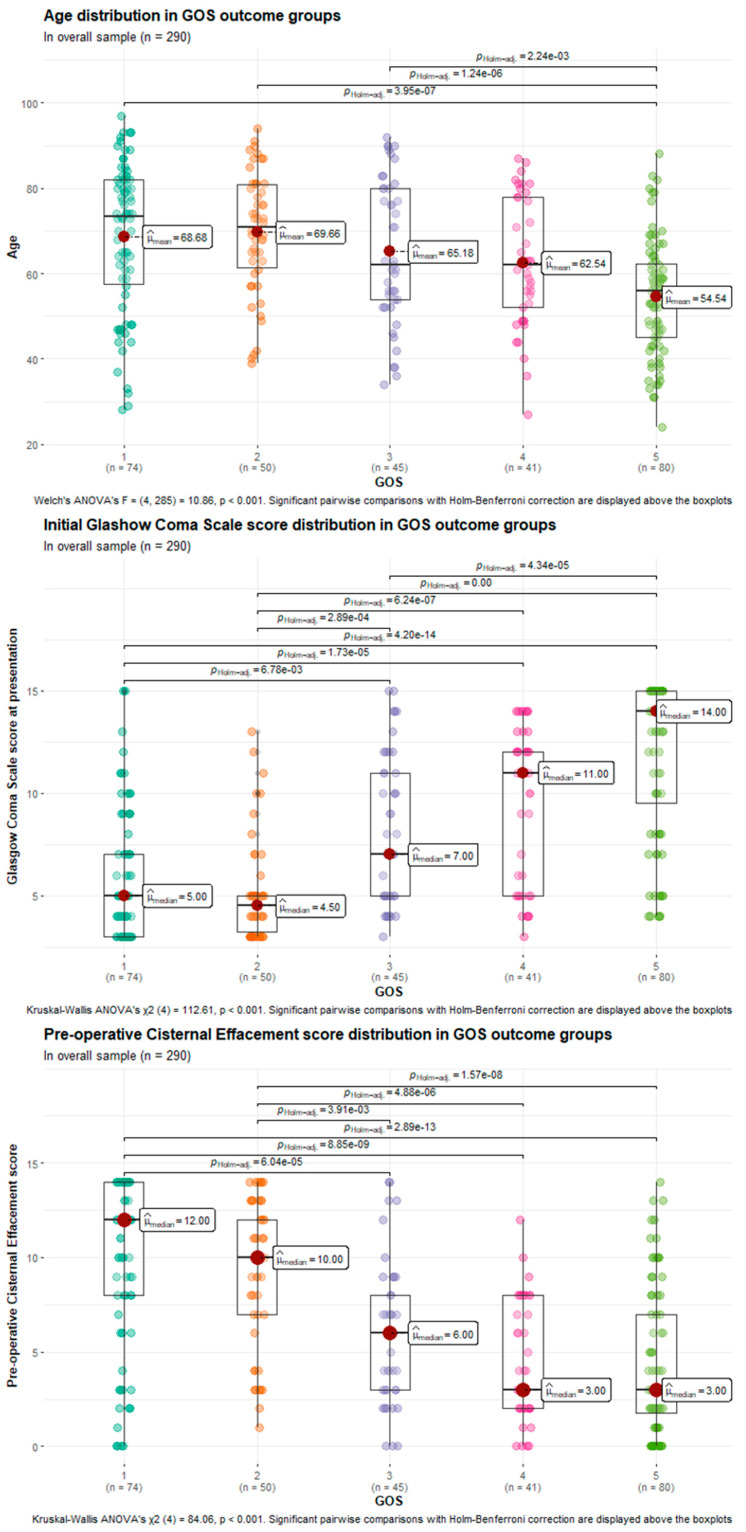
The boxplots representing the results of one-way ANOVA tests, comparing age, initial GCS score, and pre-operative CES between different GOS outcome groups in a whole sample. In addition, the figure includes Holm–Benferroni-corrected significant pairwise comparisons.

The differences in age, initial GCS, and CES among different GOS categories were assessed using a one-way ANOVA test. All three models were found to be statistically significant (*p* < 0.001 for each), indicating that age, initial GCS, and pre-operative state of cisternal effacement score play a role in distinguishing GOS categories. Results of the ANOVA analyses with significant pairwise comparisons are visualized and summarized in Figure 3.

### 3.4. Comparison of pDC and OC Groups Outcomes

The distribution of baseline characteristics was compared between deceased patients who underwent OC and surviving patients in the pDC group. The surviving patients who had decompressive craniectomy on average were younger compared to the deceased patients who received osteoplastic craniotomy (mean age 55.43 ± 14.58 vs. 72.28 ± 14.63, *p* < 0.001). Nevertheless, there were no significant differences observed in terms of initial GCS score (median 4 [4–5] vs. 5 [3–9.5], *p* = 0.35), midline shift (mean 1.46 ± 0.54 and 1.44 ± 0.89, *p* = 0.91), thickness of the hematoma (mean 1.92 ± 0.75 and 2.09 ± 0.74, *p* = 0.30), and CES on presentation (median 9 [7–12] and 10 [5–13.5], *p* =0.7) between the two groups. However, the post-operative cisternal scores of survivors after primary DC were significantly lower in comparison to deceased individuals who underwent OC (median 3 [2–5] vs. 7 [2–10], *p* = 0.01).

**Figure 4:**
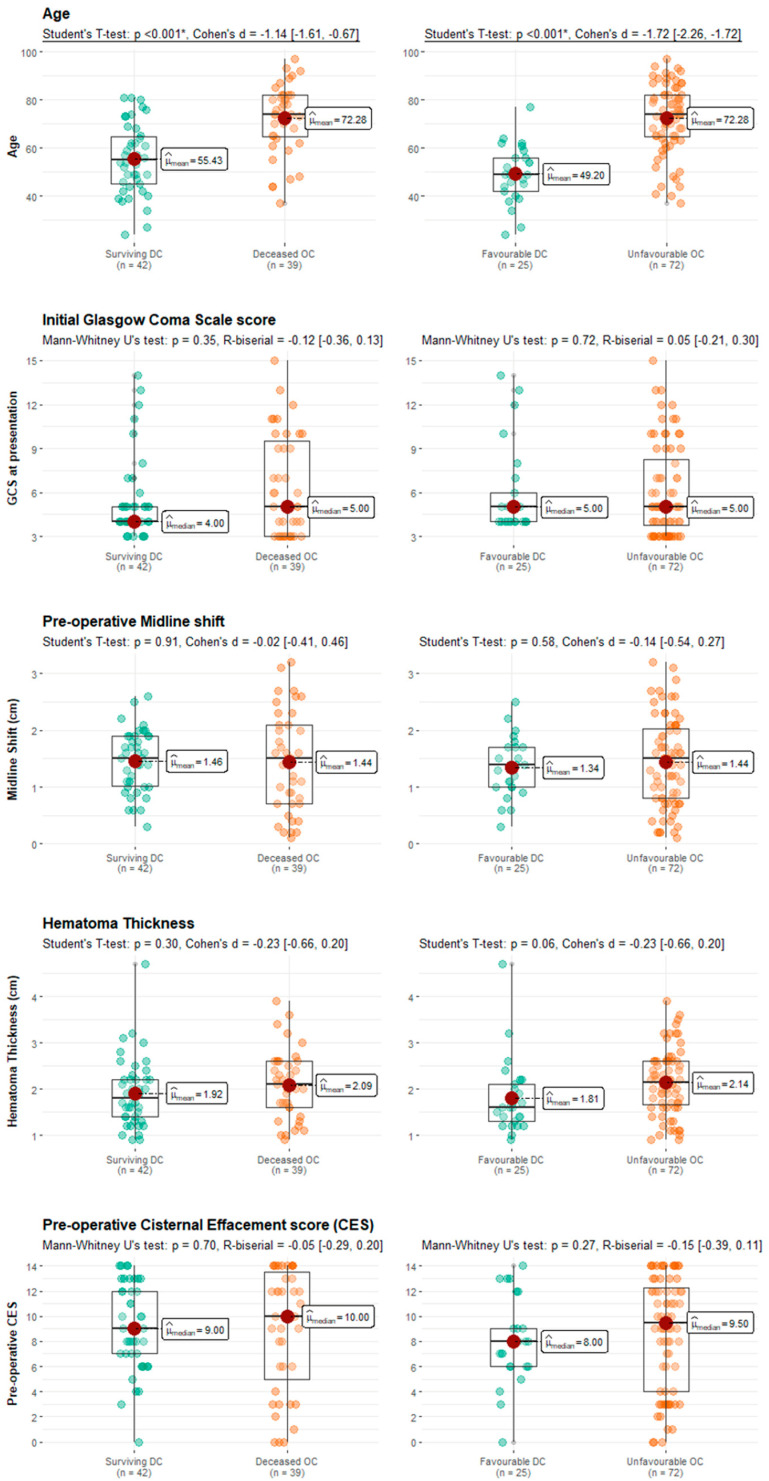
Boxplots comparing age, initial GCS, pre-operative midline shift, hematoma thickness, and CES between surviving pDC and deceased OC (left side of the graph) and favorable pDC with unfavorable OC (right half of the graph). Only age was statistically significantly different between the two groups (*p* < 0.001, Cohen’s d [95% CI) = −1.14 [−1.61, −0.67] for survived pDC and *p* < 0.001, Cohen’s d [95 CI%] = −1.72 [−2.26, −1.72] for favorable outcome pDC.

Similar results were obtained from the analysis of functional outcomes. Patients who achieved favorable outcomes and regained consciousness in the hospital after pDC were younger compared to those who had poor outcomes after OC (mean age 49.20 ± 12.05 vs. 72.28 ± 14.32, *p* < 0.001). There were no statistically significant differences between these groups in GCS score on presentation (median 5 [4–6] vs. 5 [4–8], *p* = 0.72), initial midline shift (mean 1.34 ± 0.53 and 1.44 ± 0.79, *p* = 0.58), hematoma thickness (mean 1.81 ± 0.81 and 2.14 ± 0.70, *p* = 0.06), and pre-operative CES (median 8 [6–9] and 9.5 [4–12], *p* = 0.27). Notably, the post-operative CES failed to reach statistical significance when comparing patients with favorable outcomes after pDC and individuals with unfavorable outcomes after OC (median 3 [2–7] vs. 4 [2–8], *p* = 0.09). Comparisons between successful primary DCs and unsuccessful OCs are summarized and visualized in Table 4 and Figure 4, respectively.

## 4. Discussion

The goal of this study was to identify the baseline characteristics predictive of the improved outcomes after the evacuation of aSDH in a TBI population of patients undergoing either pDC or OC. Our findings suggest that age, initial GCS score, and pre-operative degree of cisternal compression are independently associated with GOS categories in both the entire sample and the OC subgroup. However, GCS at presentation for the primary DC group failed to emerge as a predictive factor in the multivariate assessment, likely due to homogenously low GCS scores on presentation within the subgroup. Furthermore, we conducted a comparison between the cases of effective pDC and instances where OC was unsuccessful, with the aim of identifying the subpopulation of individuals who may have benefited from the decompressive surgery but instead experienced adverse outcomes such as death or persistent vegetative state following OC. The subgroup that underwent successful pDC as compared to failed OC had similar GCS scores, hematoma thickness, midline shift, and degree of cisternal compression. However, it was noted that improved outcomes were observed among younger individuals, suggesting that decompressive surgery may be most effective for younger, deeply comatose patients with a significant intracranial mass effect (Figure 4).

It is now widely accepted that decompression becomes necessary when there is no possibility to safely close the dura mater without the tension after the evacuation of aSDH. According to the consensus statement, if the brain is bulging beyond the inner table of the skull after removal of aSDH, it is advisable to leave the bone flap out [1]. Although pDC is often performed when the bone flap cannot be restored without cerebral compression, the literature remains inconclusive on the topic of pre-emptive decompression in anticipation of post-operative swelling. Many answers were expected from the international, multicentre, randomized RESCUE-ASDH trial, where patients were randomized into the pDC or OC treatment arms intraoperatively following the evacuation of aSDH [10]. Individuals where safe restoration of bone was not possible were excluded. Unfortunately, no significant difference was found between the two surgical methods in terms of functional outcomes and quality of life-related measures. Although decompressive craniectomy is most commonly performed on comatose patients, it is worth noting that one-third of the trial patients had a GCS score greater than 8. However, due to randomization, this factor should not influence the validity of the results [10,11].

Similarly, observational studies comparing outcomes after pDC and OC have yielded mixed results. Ruggeri et al. reported that patients who underwent DC had better neurological outcomes compared to those who underwent craniotomy, but there was no statistically significant difference in modified Rankin Scale scores after 12 months [12]. Castano-Leon et al. found that patients with an unfavorable prognosis achieved better-than-expected outcomes when undergoing pre-emptive decompressive craniectomy rather than craniotomy [13]. Jasielski et al. observed the best results in patients aged under 50 years, particularly those under 35 years old, and recommended early and sufficiently large decompressive craniectomy [14] Geographic areas with limited access to advanced medical treatment for severe TBI have shown potential benefits from decompressive craniectomy when performed within 5 h of injury in younger patients with a GCS score greater than 5 [15]. Zh. Lan et al. identified lower GCS, bilaterally dilated pupils, surgery performed more than 1 h after injury, and advanced age as independent risk factors for poor outcomes. They advocated for DC in subdural and intracerebral hematoma patients with GCS scores of 6 or less [16]. Soukiasian et al. did not find the choice of surgical method to be a significant predictor of death, even when adjusted for the severity of injury [17]. Woertgen et al. reported that age and clinical signs of herniation were significantly associated with unfavorable outcomes, regardless of the surgical approach [18]. However, the decision between decompression or restoration of the bone ultimately depends on the operating neurosurgeon and the specific circumstances of each case.

Primary DC is most often performed for clinical and radiographic evidence of herniation, rather than for refractory ICP elevation. Several studies have investigated the factors influencing outcomes after the evacuation of aSDH and the use of decompressive craniectomy. Yatsushige et al. found that younger age and preserved pupillary reactivity upon admission were associated with favorable outcomes [19]. Tang et al. identified predictors of 30-day mortality in patients undergoing pDC, including age, bilateral unreactive pupils, presence of completely effaced basal cisterns, intraoperative hypotension, preoperative activated partial thromboplastin time, and Injury Severity Score [20]. Lemcke et al. associated unfavorable long-term outcomes with age, midline shift, and worse status of basal cisterns on CT [21]. Juskys et al. found that worse outcomes were independently associated with older age and degree of basal cistern compression, especially in patients older than 70 years and pre-operative CES of 9 or more [22]. Performing a decompressive craniectomy may not yield a significant benefit after a certain age limit such as a 30–50-year threshold according to many authors. Patients over 65 years of age tend to do worse than their younger counterparts, while younger patient groups (<40 vs. 40–65 years of age) show no difference in outcomes measured. Based on the literature, the main factors influencing the outcomes after aSDH evacuation include age, comorbidities, severity of TBI, associated injuries, CT findings, and signs of herniation, such as degree of basal cisterns compression and pupillary state [19,20,21,22,23,24,25,26,27,28,29].

According to our research findings, the key baseline factors that significantly influence the GOS outcome include age, initial GCS score, and degree of basal cistern compression, as assessed by a multivariate assessment. While these pre-operative characteristics may be prognostic, their utility in selecting patients for pDC is limited. Therefore, we broadened our focus by comparing patients who underwent aSDH evacuation through pDC and survived or had a favorable outcome with those who did not survive or experienced an unfavorable outcome following OC. Both groups were similar in terms of GCS scores of 4–6, midline shifts of 1 to 2 cm, hematoma thickness ranging from 1 to 2.5 cm, and CES in the range of 7–12. However, successful outcomes after pDC were observed in younger individuals (mean age of 55.43 ± 14.58 and 49.20 ± 12.05 for survival and good functional recovery, respectively).

Although determining precise pre-operative thresholds to indicate pre-emptive decompression following aSDH evacuation is challenging, our data suggest that younger (<55 years old), deeply comatose (GCS 4–6) patients who meet salvageable pre-operative neuroradiological criteria (hematoma thickness 1 to 2.5 cm; midline shift 1 to 2 cm; CES in a range of 7–12) should undergo pre-emptive DC. Nonetheless, the proposed, restricted pre-operative indications for pDC need to be assessed in a prospective manner to validate the efficacy. In addition, future research should focus on identifying additional clinical and neuroimaging factors that could assist in selecting patients who are likely to benefit from decompressive surgery.

It is important to acknowledge the limitations of our study. The findings reflect a single-center experience based on retrospective analysis of the prospectively maintained TBI database. To diminish the risk of observer bias, separate researchers were assigned to data collection and analysis. The dataset lacks information on important variables, such as pupillary reactivity, door-to-surgery time, associated injuries, and pre-hospital care. Furthermore, there is a lack of long-term outcome data following TBI, as the assessments included only covered the intrahospital outcomes. To mitigate the risk of bias in our future work—additional researchers are recruited to maintain the TBI database who would be blinded to patient outcomes. Patient outcomes would be acquired in the outpatient clinic in 6 months and 2 years post-TBI time intervals. Special attention would be attributed to cognitive and life quality outcomes for TBI survivors. Another problem is the external validation of our findings that for this moment is underexplored. An external validation project of our findings is still ongoing with another TBI patient population from a different Lithuanian region.

The confirmation of already known variables that influence TBI outcomes after aSDH removal is one of the strengths of this study. In combination with radiological parameters, our study provides a means for further TBI statistical modeling, especially for the patients who could benefit from pre-emptive decompression.

## 5. Conclusions

Younger patients (<55 years old) with initial GCS scores of 4–6, midline shifts of 1 to 2 cm, hematoma thickness of 1 to 2.5 cm, and CES in a range of 7–12 may benefit from pDC as it could potentially improve survival and functional outcomes after aSDH evacuation in this subgroup of individuals.

## Figures and Tables

**Table 1:** Summary of the sample characteristics.

Variable	Overall Sample (*n* = 290)	DC Subgroup (*n* = 77)	OC Subgroup (*n* = 213)	*p*-Value
Age	63.53 ± 16.55	59.62 ± 17.63	64.95 ± 15.95	**0.02**
Preoperative GCS score	7 (4–12)	4 (3–5)	10 (5–14)	**<0.001**
Hematoma Thickness (mm)	18.7 ± 6.6	20.1 ± 7.3	18.2 ± 6.3	0.052
Preoperative Midline Shift (mm)	11.6 ± 7.2	15.4 ± 6.8	10.2 ± 6.9	**<0.001**
Post-operative Midline Shift (mm)	5.7 ± 4.6	6.1 ± 5.9	5.5 ± 4.1	0.43
Preoperative cisternal effacement score	7 (3–11)	11 (8–13)	4 (2–9)	**<0.001**
Post-operative cisternal effacement score	2 (1–6)	4 (2–11)	2 (0–4)	**0.003**

**Table 2:** The results of the univariate analysis examining the relationship between pre-operative factors and GOS categories using a proportional odds logistic regression method.

Characteristic	Overall Sample (*n* = 290)		DC Subgroup (*n* = 77)		OC Subgroup (*n* = 213)	
	OR (95% CI)	*p*-Value	OR (95% CI)	*p*-Value	OR (95% CI)	*p*-Value
Age	0.96 (0.95–0.97)	**<0.001**	0.96 (0.93–0.98)	**<0.001**	0.95 (0.93–0.96)	**<0.001**
Preoperative GCS score	1.36 (1.28–1.45)	**<0.001**	1.17 (0.99–1.38)	0.074	1.37 (1.28–1.47)	**<0.001**
Hematoma Thickness	0.38 (0.27–0.53)	**<0.001**	0.54 (0.28–0.99)	**0.047**	0.35 (0.23–0.52)	**<0.001**
Preoperative Midline Shift	0.3 (0.22–0.41)	**<0.001**	0.57 (0.29–1.08)	0.084	0.31 (0.21–0.46)	**<0.001**
Preoperative cisternal effacement score	0.78 (0.74–0.82)	**<0.001**	0.80 (0.70–0.91)	**<0.001**	0.8 (0.75–0.85)	**<0.001**

**Table 3:** The results of multivariate proportional odds logistic regression identifying the pre-operative characteristics independently associated with ordinal GOS categories. The goodness-of-fit of the model was assessed using the Lipsitz test, confirming the assumption of proportional odds (*p* = 0.46 for a whole sample, *p* = 0.23 for pDC, and *p* = 0.35 for OC subgroups, respectively).

Characteristic	Overall Sample (*n* = 290)		pDC Subgroup (*n* = 77)		OC Subgroup (*n* = 213)	
	OR (95% CI)	*p*-Value	OR (95% CI)	*p*-Value	OR (95% CI)	*p*-Value
Age	0.96 (0.93–0.96)	**<0.001**	0.95 (0.92–0.98)	**<0.001**	0.94 (0.92–0.96)	**<0.001**
Preoperative GCS score	1.29 (1.20–1.39)	**<0.001**	1.08 (0.90–1.30)	0.40	1.31 (1.20–1.42)	**<0.001**
Hematoma Thickness	0.98 (0.63–1.52)	0.98	0.50 (0.20–1.10)	0.088	1.31 (0.74–2.36)	0.41
Preoperative Midline Shift	1.05 (0.61–1.81)	0.90	1.81 (0.65–5.46)	0.32	0.84 (0.43–1.66)	0.65
Preoperative cisternal effacement score	0.85 (0.79–0.92)	**<0.001**	0.78 (0.66–0.91)	**0.002**	0.89 (0.81–0.97)	**<0.001**

**Table 4:** Comparison of successful pDCs and unsuccessful OCs in terms of mortality and functional outcome.

	Mortality				Functional Outcome			
	Overall (*n* = 81)	Surviving DC (*n* = 42)	Deceased OC (*n* = 39)	*p*-Value	Overall (*n* = 97)	Favorable DC (*n* = 25)	Unfavorable OC (*n* = 72)	*p*-Value
Age	63.54 ± 16.81	55.43 ± 14.58	72.28 ± 14.63	**<0.001**	66.33 ± 17.05	49.20 ± 12.05	72.28 ± 14.32	**<0.001**
Pre-op GCS	5 (4–7)	4 (4–5)	5 (3–9.5)	0.35	5 (4–7)	5 (4–6)	5 (4–8)	0.72
Pre-op Midline Shift (cm)	1.45 ± 0.72	1.46 ± 0.54	1.44 ± 0.89	0.91	1.41 ± 0.73	1.34 ± 0.53	1.44 ± 0.79	0.58
Hematoma Thickness (cm)	2.00 ± 0.75	1.92 ± 0.75	2.09 ± 0.74	0.30	2.05 ± 0.74	1.81 ± 0.81	2.14 ± 0.70	0.06
Pre-op cisternal effacement score	9 (6–13)	9 (7–12)	10 (5–13.5)	0.70	9 (6–12)	8 (6–9)	9.5 (4–12)	0.27

## Data Availability

The data are not publicly available due to ethical restrictions by the national ethical committee regulations.
